# Antibody in Lymphocyte Supernatant (ALS) responses after oral vaccination with live *Shigella sonnei* vaccine candidates WRSs2 and WRSs3 and correlation with serum antibodies, ASCs, fecal IgA and shedding

**DOI:** 10.1371/journal.pone.0259361

**Published:** 2021-11-18

**Authors:** Malabi M. Venkatesan, Cassandra Ballou, Shoshana Barnoy, Monica McNeal, Jill El-Khorazaty, Robert Frenck, Shahida Baqar

**Affiliations:** 1 Bacterial Diseases Branch, Walter Reed Army Institute of Research, Silver Spring, MD, United States of America; 2 The Emmes Company, LLC, Rockville, MD, United States of America; 3 Division of Infectious Diseases, Department of Pediatrics, University of Cincinnati College of Medicine, Cincinnati Children’s Hospital Medical Center, Cincinnati, OH, United States of America; 4 Division of Microbiology and Infectious Diseases, National Institute of Allergy and Infectious Diseases, National Institutes of Health, Bethesda, Maryland, United States of America; Instituto Butantan, BRAZIL

## Abstract

The levels of antigen-specific Antibodies in Lymphocyte Supernatant (ALS) using an ELISA are being used to evaluate mucosal immune responses as an alternate to measuring the number of Antibody Secreting Cells (ASCs) using an ELISpot assay. A recently completed trial of two novel *S*. *sonnei* live oral vaccine candidates WRSs2 and WRSs3 established that both candidates were safe, well tolerated and immunogenic in a vaccine dose-dependent manner. Previously, mucosal immune responses were measured by assaying IgA- and IgG-ASC in peripheral blood mononuclear cells (PBMCs). In this report, the magnitude of the *S*. *sonnei* antigen-specific IgA- and IgG-ALS responses was measured and correlated with previously described ASCs, serum antibodies, fecal IgA and vaccine shedding. Overall, the magnitude of *S*. *sonnei* anti-Invaplex50 ALS was higher than that of LPS or IpaB, and both vaccines demonstrated a more robust IgA-ALS response than IgG; however, compared to WRSs3, the magnitude and percentage of responders were higher among WRSs2 recipients for IgA- or IgG-ALS. All WRSs2 vaccinees at the two highest doses responded for LPS and Invaplex50-specific IgA-ALS and 63–100% for WRSs3 vaccinees responded. Regardless of the vaccine candidate, vaccine dose or detecting antigen, the kinetics of ALS responses were similar peaking on days 7 to 9 and returning to baseline by day 14. The ALS responses were vaccine-specific since no responses were detected among placebo recipients at any time. A strong correlation and agreement between responders/non-responders were noted between ALS and other mucosal (ASC and fecal IgA) and systemic (serum antibody) immune responses. These data indicate that the ALS assay can be a useful tool to evaluate mucosal responses to oral vaccination, an observation noted with trials of other bacterial diarrheal pathogens. Furthermore, this data will guide the list of immunological assays to be conducted for efficacy trials in different populations. It is hoped that an antigen-specific-ALS titer may be a key mucosal correlate of protection, a feature not currently available for any *Shigella* vaccines candidates. https://clinicaltrials.gov/show/NCT01336699.

## Introduction

*Shigella* continues to be a cause of significant morbidity and mortality in the world, particularly in young children living in low to medium income countries [1,2]. In sub-Saharan Africa, Shigella was the second leading cause of mortality due to diarrheal diseases among all ages [3]. An additional concern limiting treatment options is the evolution of multidrug-resistant *Shigella* strains. Thus, control measures have primarily focused on development of vaccines that include whole -cells killed, live attenuated and various subunit-based *Shigella* vaccines [4–7]. Following vaccination or infection the ability to measure the immune response, using reproducible and technologically simple methods is critical, particularly if evaluating a vaccine candidate in a resource limited region.

Previous clinical studies with live oral, *virG(icsA)-*based *Shigella* vaccine candidates relied on determinations of IgA/IgG serum antibodies and antibody secreting cells (ASC) in peripheral blood mononuclear cells (PBMCs) as one of the primary mucosal immune response measures [8–12]. The ASC response uses an ELISPOT assay for the direct measurement of antibody producing cells at the cellular level in a solid phase format [13]. Although ELISPOT detects the actual number of B or plasma cells secreting antigen-specific antibodies, the requirement for large number of PBMCs per antigen limits its utility to investigate responses against several antigens and isotypes. Investigators are seeking ways to bring immunological evaluation of candidate vaccines to the site of vaccine testing. The ASC assay may be difficult to transfer to resource-limited settings, hence the detection of Antibodies in Lymphocyte Supernatant (ALS) by ELISA is considered an attractive alternate and has been used in other bacterial vaccine-related studies [14–23]. Both ASC and ALS assays utilize PBMCs and the kinetics of responses by both methods are similar. However, in contrast to the ASC assay, the ALS assay detects the total amount of antibody secreted by mucosally-activated PBMCs cultured *ex-vivo* in a liquid phase. This provides a larger volume of analyzable antibody-enriched supernatant which can be stored and used to determine responses to multiple antigens and/or isotypes, increasing the flexibility and versatility of the assay [13–23].

The recent placebo-controlled phase 1 trial of two *S*. *sonnei* vaccine candidates, WRSs2 and WRSs3, provided the opportunity to directly compare the immune responses measured by ALS to that of previously described ASC and serum IgG and IgA as a potential bridge to ALS replacing ASC in future oral *Shigella* vaccine clinical trials [8]. The primary attenuating feature of both candidates is the loss of the invasion plasmid-encoded *Shigella virG* (or *icsA*) gene, whose product facilitates intercellular bacterial spread after invasion of epithelial cells [24,25]. Additionally, both candidates lacked the virulence plasmid-encoded enterotoxin gene *senA* and its paralog *senB* [24,25]. WRSs3 also lacks the virulence plasmid-encoded *msbB2* gene that is required for maximal LPS endotoxicity [25]. Samples were collected periodically to determine vaccine strain shedding and immune responses to *Shigella* antigens. Both candidates were safe, well tolerated, and immunogenic in a vaccine dose-dependent manner [8]. Immunogenicity data in the form of serum IgA and IgG and IgA- and IgG-ASC responses have been described earlier [8]. Here we report in detail the mucosal response to WRSs2 and WRSs3 vaccination as measured by the antigen-specific IgA- and IgG-ALS, a feature not previously reported for the *virG*-based live vaccine candidates. We examined the correlations between the ALS responses and other previously described immune measurements such as ASCs (IgA, IgG), fecal IgA, serum antibodies, and with vaccine shedding (8). Such a detailed analysis of ALS with other immune responses will provide opportunities following an efficacy trial to establish correlates of mucosal protection.

## Materials and methods

### Vaccine and vaccination

The details of the phase 1 clinical trial of WRSs2 and WRSs3 vaccine candidates, sample collection and assay procedures have been previously described [8]. The study was reviewed and approved by the Cincinnati Children’s Hospital Medical Center IRB, FWA00002988. Briefly, novel *virG(icsA)*-based live, attenuated *S*. *sonnei* vaccine candidates were delivered as a single oral dose of 10^3^−10^7^ CFU to 8 subjects/dose and 9 subjects received saline placebo. All subjects were healthy adults who provided written informed consent in front of two witnesses. Serum, PBMCs, and stool for fecal IgA and shedding were collected and stored until assayed (8). Stool samples were collected at least daily beginning on the day of admission to the inpatient unit through the day of discharge on day 9. Additionally, if the subject was experiencing diarrhea, upto one additional sample per 8-hour shift was collected. Stool for culture also was obtained on day 14 and 28 post vaccination.

### Sample collection for ALS assay and processing

Peripheral blood samples were collected pre-vaccination and 7, 9, and 14 days post-vaccination, PBMCs were isolated and stored in liquid nitrogen until used. The cryopreserved PBMCs were thawed (average viability after thawing ~80%) and cultured *in vitro* for 72 hours at a density of 1 X 10^7^ cells per mL in RPMI, with 10% fetal bovine serum, penicillin and streptomycin and glutamine (all reagents from Thermo Fisher) at 37°C and 5% CO2 for 4 days. Supernatants were harvested and frozen at -80°C until testing [8]. The ALS assays were carried out once all samples from every dose had been collected.

### ALS-ELISA assay

The stored supernatants were used to measure antigen-specific antibodies by ELISA against *S*. *sonnei* LPS, *S*. *sonnei* Invaplex50 (IVP; ion-exchange extract from virulent *S*. *sonnei* that consists of a mixture of *S*. *sonnei* LPS and IpaB and IpaC as well as several other minor proteins) and purified IpaB protein. Details of the ELISA procedure has been published previously [8]. Briefly, ELISA plates (Thermo Fisher) were coated overnight with each antigen at 1μg/well, 0.05μg/well or 0.0125 μg/ well for LPS, IVP and IpaB proteins respectively, in PBS and blocked with 2% casein in Tris-NaCl buffer. Dilutions of each supernatant samples in 2% casein buffer were added in duplicate to each antigen wells. Following 2 hours incubation, plates were washed with PBS + 0.05% Tween and ALK-P-conjugated goat anti-human IgA or IgG were added and incubated for 1 hour. Plates were then washed, and bound antibody was detected using pNPP substrate (Sigma Aldrich) in diethanolamine buffer. The optical density was measured at 405nm and the endpoint titer was determined as the reciprocal of the highest dilution of sample with an average OD ≥0.2.

### Statistical analysis

The phase 1 study was not powered to detect statistical significance between two vaccine candidates or to detect a significant correlation between assays. Therefore, the analysis presented here focus on estimates and confidence intervals to describe the ALS response among this group of subjects as well as the association between ALS and other immune measurements and fecal shedding.

For all antibody measurements by ELISA such as IgA- and IgG-ALS, serum IgA or IgG, and fecal IgA, response was defined as the raw value of the titer at a given time point. A responder was defined as having a ≥4 fold increase in titer over baseline. For IgA- and IgG-ASC, response was defined as the raw number of ASC/10^6^ PBMCs and a responder was defined as a subject with ≥10 ASC/10^6^ PBMCs. For computing summary statistics and log transformations, ASC values of zero were imputed with a value of 0.5 and for log transformations value of zero were imputed with a value of 2.5. For vaccine shedding, a response was defined as maximum post-baseline CFU/gm of stool and a responder was defined as a subject with maximum of >0 CFU/gm of stool.

The magnitude of the ALS response was summarized by determining the maximum fold increase for each subject and computing the geometric mean (GM) and the geometric standard deviation (GSD) for each vaccine candidate dose-group.

Pearson correlations between the various mucosal, serological, and vaccine shedding responses were computed using maximum of the log_10_ transformed titers as the continuous variable with the corresponding 95% confidence interval (Cl).

Responses were dichotomized into responders and non-responders as described above. Agreement of responders/non-responders between assays was assessed using kappa statistics presented with 95% confidence intervals in addition to cross tabulations showing the number and percentage of responders/non-responders of ALS with other mucosal and serological responses and vaccine shedding. All analyses were performed using SAS version 9.4.

## Ethical review

The study was reviewed and approved by the CCHMC IRB FWA00002988 and conducted according to the standards of ICH-GCP E6, under a US Food and Drug Administration-approved IND.

The investigators have adhered to the policies for protection of human subjects as prescribed in AR 70–25.

## Results

### Study subjects

A total of 89 subjects participated in the study, 5 cohorts of 8 subjects each received either a dose of WRSs2 (n = 40) or WRSs3 (n = 40) and 9 subjects received placebo [8]. The baseline immunological characteristics of the subjects by groups showed that based on comparing estimates and measures of dispersion, baseline values between vaccinee groups and placebos are very similar (data not shown).

### Magnitude of the ALS response

**Fig 1:**
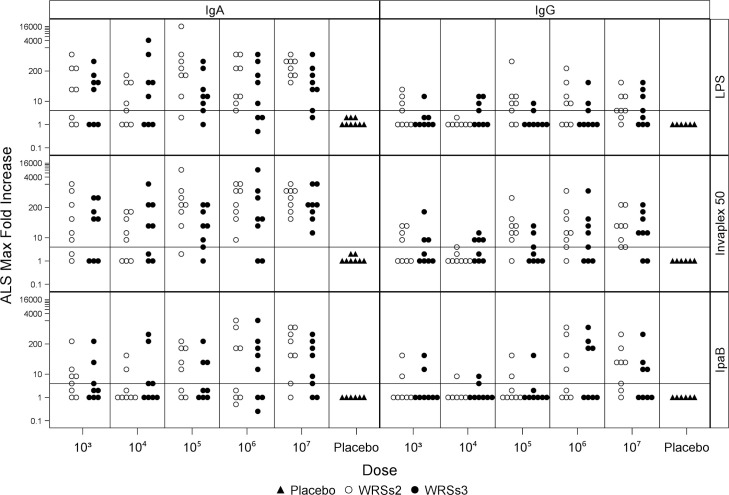
ALS maximum fold increase per cohort. The data are presented as the maximum fold increase of ALS IgA/IgG titer over baseline for each subject in each vaccine dose group. The open circles represent WRSs2 vaccinated subjects, the closed circles represent WRSs3 vaccinated subjects and the triangles represent placebo-immunized subjects. The Y-axis gives the maximum fold increase of IgA (left panel) and IgG (right panel) ALS titer of individual subjects to *S*. *sonnei* antigens LPS, IVP and IpaB shown on the X axis (CFU doses of the vaccine). The thin horizontal line shows the level of the 4-fold increase that defined a responder.

PBMCs for the ALS assay were collected prior- to and 7, 9 and 14 days post-vaccination. The ALS maximum fold increase for each vaccinee in each cohort is depicted in Fig 1 where a responder can be identified as a dot above the 4-fold increase line. The geometric mean of the maximum fold increase in antigen-specific IgA- and IgG- ALS and the responder rates for each vaccine dose and candidate is summarized in Table 1. The estimates of the magnitude of IgA-ALS responses to all three antigens are numerically higher than that of the IgG-ALS responses in both groups of vaccinated subjects. The IgA-ALS maximum fold increase for IVP was numerically higher than for LPS and IpaB and overall, the IgA-ALS responses for WRSs2 vaccinees reached higher levels than with WRSs3 vaccinees. Notably, there were no IgA- and IgG-ALS responders to any of the *Shigella* antigens among the placebos. At the two highest doses (10^6^ and 10^7^ CFU) all subjects receiving WRSs2 had at least a 4-fold rise in LPS- and IVP-specific IgA-ALS titers. At the same doses, more than 63% of the subjects vaccinated with WRSs3 responded with a 4-fold rise to both antigens with all subjects responding to IVP at 10^7^ CFU. Additionally, ≥50% of the subjects in both vaccine groups responded with an IpaB-specific IgA- and IgG-ALS response at the two highest doses (Table 1). While a vaccine dose-dependent increase was seen in IgA-ALS responses, but not in IgG responses, particularly to IVP and IpaB in WRSs2 vaccinees and to IVP in WRSs3 vaccines, in both groups of vaccinees, the magnitude of the IgA and IgG responses to all 3 antigens were highest at the 10^7^ CFU dose (Fig 1 and Table 1**).** There were more subjects in the WRSs2-vaccinated group with an IgG response to all 3 antigens as compared to the WRSs3 group, although the magnitude of the IgG-ALS response was similar for both vaccine candidates (Fig 1 and Table 1).

**Table 1:** Magnitude of the ALS response in WRSs2 and WRSs3 vaccinees: IgA and IgG to LPS, IVP and IpaB.

Treatment and dose (cfu)		Maximum fold increase from baseline following vaccination; GM ± GSD (% responders)					
		IgA-ALS			IgG-ALS		
Vaccine	Dose	LPS	Invaplex	IpaB	LPS	Invaplex	IpaB
**WRSs2**	**10** ^**3**^	24.7 ± 15.2 (63)	41.5 ± 16.8 (75)	6.2 ± 6.1 (63)	3.4 ± 4.1 (50)	4.4 ± 5.1 (50)	2.2 ± 4.7 (25)
	**10** ^**4**^	8.0 ± 7.9 (63)	10.4 ± 8.7 (63)	2.6 ± 5.0 (25)	1.1 ± 1.3 (0)	1.3 ± 1.7 (13)	1.3 ± 2.2 (13)
	**10** ^**5**^	181.0 ± 14.8 (88)	197.4 ± 11.7 (88)	16.0 ± 9.8 (63)	8.7 ± 7.2 (75)	22.6 ± 5.9 (88)	2.4 ± 4.6 (25)
	**10** ^**6**^	69.8 ± 9.4 (100)	304.4 ± 7.0 (100)	20.7 ± 29.8 (50)	8.0 ± 7.9 (63)	22.6 ± 8.7 (88)	13.5 ± 18.0 (50)
	**10** ^**7**^	279.2 ± 2.6 (100)	469.5 ± 3.3 (100)	83.0 ± 13.0 (88)	6.2 ± 3.8 (75)	26.9 ± 5.2 (100)	19.0 ± 8.3 (75)
**WRSs3**	**10** ^**3**^	17.4 ± 12.2 (63)	24.7 ± 16.0 (63)	4.4 ± 7.5 (38)	1.7 ± 2.6 (13)	3.4 ± 5.6 (38)	2.4 ± 5.2 (25)
	**10** ^**4**^	26.9 ± 25.1 (63)	26.9 ± 17.5 (63)	6.2 ± 13.4 (50)	3.1 ± 3.6 (50)	3.4 ± 3.2 (50)	1.5 ± 2.3 (25)
	**10** ^**5**^	20.7 ± 7.9 (88)	26.9 ± 7.5 (88)	5.7 ± 8.4 (38)	1.5 ± 2.3 (25)	2.8 ± 4.0 (38)	1.8 ± 4.3 (13)
	**10** ^**6**^	22.6 ± 16.8 (63)	69.8 ± 23.9 (75)	19.0 ± 23.6 (63)	2.6 ± 4.6 (38)	10.4 ± 12.0 (63)	16.0 ± 20.8 (50)
	**10** ^**7**^	45.3 ± 7.9 (88)	234.8 ± 5.1 (100)	20.7 ± 12.0 (75)	4.8 ± 5.4 (50)	17.4 ± 7.7 (75)	6.7 ± 10.0 (50)
**Placebo**	**none**	1.3 ± 1.4 (0)	1.2 ± 1.4 (0)	1.0 ± 1.0 (0)	1.0 ± 1.0 (0)	1.0 ± 1.0 (0)	1.0 ± 1.0 (0)

The data represents the geometric mean (GM) and geometric standard deviation (GSD) of the maximum fold increase in end-point titers from baseline of *S*. *sonnei* antigen-specific IgA and IgG ALS for both vaccine candidates. The percentage of responders (maximum fold increase ≥4) is given in parentheses.

### Kinetics of the ALS responses

**Fig 2:**
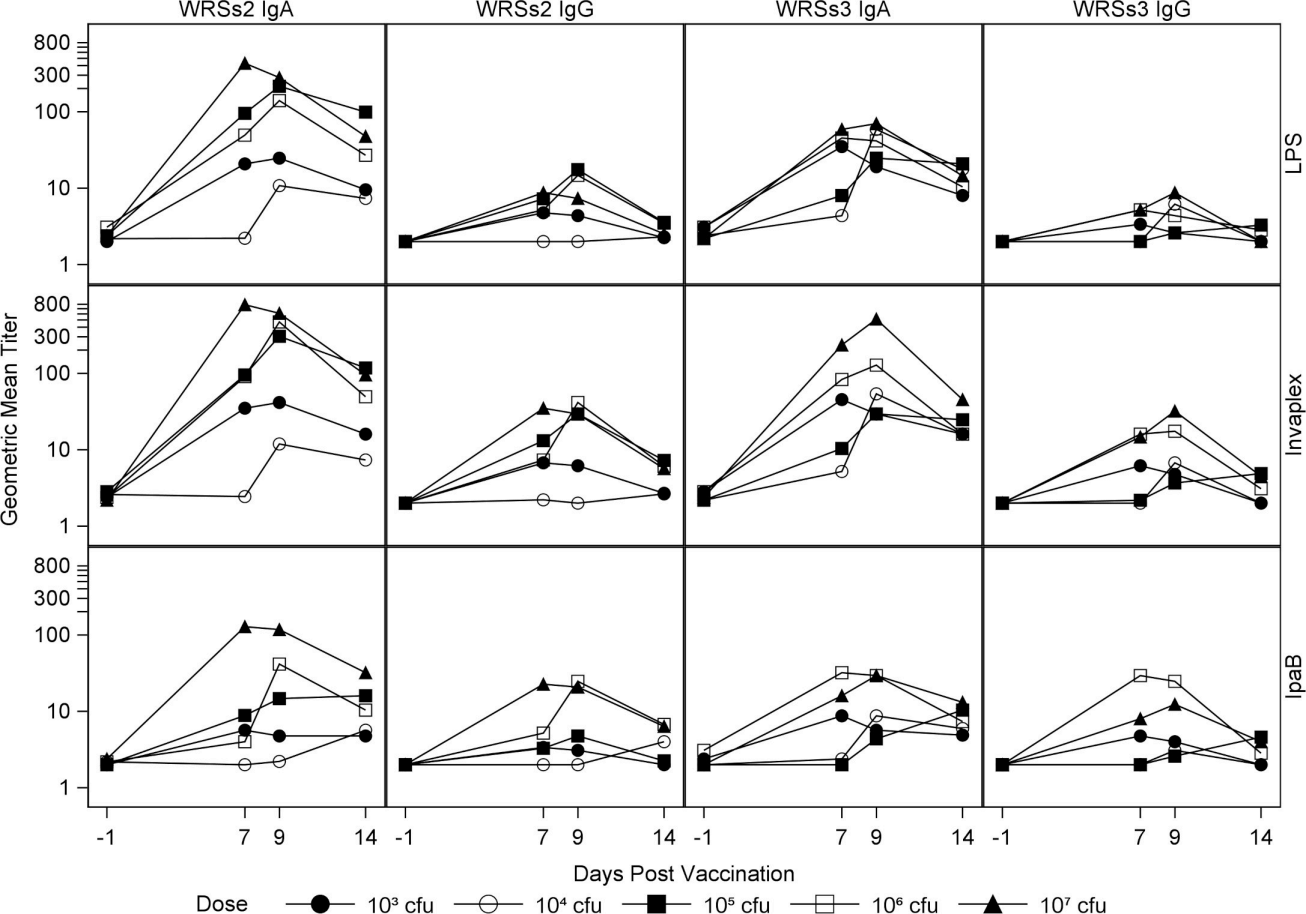
Kinetics of ALS responses following vaccination with WRSs2 and WRSs3. The Y axis data represents the geometric mean titer (GMT) of the raw ALS titer values at Day -1, Day 7, Day 9, and Day 14. For each *S*. *sonnei* antigen, LPS, IVP and IpaB in each cohort (cohorts marks: closed circle-10^3^ CFU, open circle-10^4^ CFU, closed square- 10^5^ CFU, open square- 10^6^ CFU and closed triangle- 10^7^ CFU). The X axis shows the day post-vaccination when ALS was measured.

The kinetics of the IgA- and IgG-ALS responses, irrespective of the detecting *Shigella* antigen were similar, however, IgA-ALS geometric mean titers were mostly higher than IgG (Fig 2). Both IgA- and IgG-ALS responses increased and peaked and/or plateaued around day 7–9, thereafter declining or returning to baseline levels by day 14 (Fig 2).

### Correlation between ALS and other immune responses

When examining the correlation between the maximum ALS response to that of other assays, the strongest correlation with both vaccine candidates was observed between antigen-specific IgA- and IgG-ALS and the corresponding ASC, as well as between ALS and serum IgA and IgG antibodies (correlation coefficient ≥0.66; lower 95% Confidence Limit (LCL) ≥0.52) (Table 2). The correlation between IgA ALS and fecal IgA was weaker with the LCL >0.38 across antigens. There was no correlation between ALS and shedding with WRSs2 (Table 2). A strong correlation (correlation coefficient (≥0.48, LCL>0.19) also exists between IgA- and IgG-ASCs and serum IgA and IgG for all 3 antigens in both groups of vaccines. For some measurements, the correlation was vaccine candidate dependent, for instance correlation of vaccine shedding with several immune categories of responses was only seen among WRSs3 vaccine recipients (Table 2**).** These included correlation of shedding with IgA/IgG-ALS responses to IVP, with IgA/IgG ASCs to LPS and IVP, with serum IgA to IVP and with fecal IgA to LPS and IpaB.

**Table 2:** Correlation among systemic and mucosal immune responses in WRSs2 and WRSs3 vaccinees.

Antibody Isotype and immune responses measured by assays			Pearson Correlation (95%CI)					
			WRSs2			WRSs3		
			LPS	Invaplex	IpaB	LPS	Invaplex	IpaB
**IgA**	**ALS**	**ASC**	0.77 (0.60, 0.87)	0.80 (0.65, 0.89)	0.82 (0.68, 0.90)	0.62 (0.38, 0.78)	0.86 (0.74, 0.92)	0.80 (0.66, 0.89)
		**Fecal**	0.55 (0.29, 0.73)	0.58 (0.33, 0.75)	0.52 (0.25, 0.71)	0.48 (0.20, 0.69)	0.73 (0.54, 0.85)	0.71 (0.52, 0.84)
		**Serum**	0.76 (0.58, 0.86)	0.71 (0.52, 0.84)	0.86 (0.74, 0.92)	0.81 (0.67, 0.90)	0.81 (0.66, 0.89)	0.87 (0.77, 0.93)
		**Shedding**	0.13 (-0.23, 0.46)	0.24 (-0.12, 0.55)	-0.04 (-0.39, 0.31)	0.42 (0.06, 0.69)	0.59 (0.28, 0.79)	0.12 (-0.27, 0.47)
	**ASC**	**Fecal**	0.41 (0.12, 0.64)	0.33 (0.02, 0.58)	0.51 (0.24, 0.71)	0.49 (0.21, 0.70)	0.64 (0.41, 0.79)	0.69 (0.48, 0.82)
		**Serum**	0.56 (0.30, 0.74)	0.48 (0.19, 0.69)	0.77 (0.60, 0.87)	0.61 (0.36, 0.77)	0.68 (0.47, 0.82)	0.71 (0.51, 0.83)
		**Shedding**	0.31 (-0.05, 0.59)	0.34 (-0.01, 0.62)	0.06 (-0.30, 0.40)	0.56 (0.23, 0.77)	0.77 (0.56, 0.89)	0.38 (0.00, 0.66)
	**Fecal**	**Serum**	0.66 (0.43, 0.80)	0.65 (0.42, 0.80)	0.68 (0.47, 0.82)	0.44 (0.15, 0.66)	0.73 (0.55, 0.85)	0.73 (0.55, 0.85)
		**Shedding**	0.21 (-0.15, 0.52)	0.15 (-0.21, 0.48)	0.13 (-0.23, 0.46)	0.41 (0.05, 0.68)	0.34 (-0.04, 0.63)	0.45 (0.09, 0.70)
	**Serum**	**Shedding**	0.04 (-0.31, 0.39)	0.13 (-0.23, 0.45)	-0.13 (-0.46, 0.23)	0.28 (-0.11, 0.59)	0.41 (0.05, 0.68)	0.22 (-0.16, 0.55)
**IgG**	**ALS**	**ASC**	0.69 (0.49, 0.83)	0.81 (0.67, 0.90)	0.85 (0.73, 0.92)	0.68 (0.47, 0.82)	0.86 (0.75, 0.92)	0.89 (0.80, 0.94)
		**Serum**	0.61 (0.37, 0.77)	0.60 (0.35, 0.76)	0.78 (0.63, 0.88)	0.66 (0.44, 0.81)	0.71 (0.51, 0.84)	0.85 (0.72, 0.92)
		**Shedding**	0.10 (-0.25, 0.44)	0.03 (-0.32, 0.38)	-0.22 (-0.54, 0.14)	0.39 (0.02, 0.67)	0.41 (0.04, 0.68)	0.10 (-0.29, 0.45)
	**ASC**	**Serum**	0.53 (0.26, 0.72)	0.50 (0.22, 0.70)	0.78 (0.62, 0.88)	0.66 (0.44, 0.81)	0.66 (0.45, 0.81)	0.79 (0.63, 0.88)
		**Shedding**	0.01 (-0.34, 0.35)	0.13 (-0.23, 0.46)	-0.07 (-0.41, 0.28)	0.51 (0.17, 0.74)	0.54 (0.20, 0.76)	0.20 (-0.19, 0.53)
	**Serum**	**Shedding**	0.03 (-0.32, 0.37)	0.11 (-0.24, 0.44)	-0.22 (-0.53, 0.14)	0.32 (-0.06, 0.62)	0.24 (-0.14, 0.57)	0.16 (-0.23, 0.50)

The correlation among the immune response measurements was carried out using Pearson correlation with 95% CI of the maximum log_10_ transformed end-point titers in *S*. *sonnei*-antigen-specific IgA- and IgG-ALS, serum antibodies and fecal IgA responses and *S*. *sonnei* antigen-specific peak IgA- and IgG-ASCs. Shedding is described as maximum vaccine shedding for each vaccinee in CFU/gm of stool.

### Agreement between ALS responders and ASC, serum antibodies, fecal IgA and shedding responders

**Table** 3 summarizes the agreement between dichotomized responders/non-responders of IgA- and IgG-ALS (n = 80) with other immunological and shedding responders/non-responders using Cohen’s Kappa statistic and associated confidence intervals. A strong agreement (Kappa >0.7, LCL ≥0.5) is seen between IgA-and IgG-ALS responders/non-responders and IgA- and IgG-ASC and IgA serum antibody responders to IVP and IpaB-specific antigens. A weaker but still high agreement (Kappa >0.55, LCL ≥0.44) was also observed between LPS-specific IgA-and IgG-ALS responders and IgA- and IgG-ASC and serum responders. A similar level of agreement is also seen between LPS and IVP-specific IgA-ALS responders and vaccine shedding (Kappa >0.63, LCL >0.47). Little to no agreement is seen between IgA-ALS and fecal IgA responders to all 3 antigens, between IpaB-specific IgA- and IgG-ALS responders and shedding, between LPS-specific IgG-ALS responders and shedding (**Table 3**). In most IgA-ALS cases of discordance (off-diagonal numbers in each matrix in **Table 3**), the positive ALS responders outnumber the positive responders to the second immune parameter (**Table 3**). For example, 9 subjects were positive for IVP-specific IgA-ALS and negative for IgA-ASC, while only one vaccinee was positive for IVP-specific IgA-ASC and negative for IgA-ALS to the same antigen.

**Table 3:** Agreement of ALS responders with other mucosal and serologic responders and fecal shedding (Kappa statistic; 95% Cl).

Isotype	Assay		LPS		Invaplex		IpaB	
			-	+	-	+	-	+
**IgA**	**ASC**	**-**	27 (30)	18 (20)	23 (26)	9 (10)	43 (48)	11 (12)
		**+**	0 (0)	44 (49)	1 (1)	56 (63)	2 (2)	33 (37)
		**Kappa (95%CI)**	**0.597 (0.445, 0.749)**		**0.742 (0.595, 0.889)**		**0.707 (0.563, 0.851)**	
	**Fecal**	**-**	17 (19)	24 (27)	18 (20)	15 (17)	29 (33)	20 (22)
		**+**	10 (11)	38 (43)	6 (7)	50 (56)	16 (18)	24 (27)
		**Kappa (95%CI)**	**0.212 (0.017, 0.406)**		**0.464 (0.273, 0.655)**		**0.190 (-0.013, 0.393**	
	**Serum**	**-**	26 (29)	14 (16)	23 (26)	10 (11)	45 (51)	12 (13)
		**+**	1 (1)	48 (54)	1 (1)	55 (62)	0 (0)	32 (36)
		**Kappa (95%CI)**	**0.649 (0.495, 0.803)**		**0.719 (0.569, 0.870)**		**0.729 (0.592, 0.867)**	
	**Shedding**	**-**	24 (27)	12 (13)	23 (26)	13 (15)	27 (30)	9 (10)
		**+**	3 (3)	50 (56)	1 (1)	52 (58)	18 (20)	35 (39)
		**Kappa (95%CI)**	**0.636 (0.472, 0.799)**		**0.655 (0.497, 0.813)**		**0.395 (0.208, 0.581)**	
**IgG**	**ASC**	**-**	49 (55)	13 (15)	33 (37)	4 (4)	54 (61)	2 (2)
		**+**	5 (6)	22 (25)	8 (9)	44 (49)	7 (8)	26 (29)
		**Kappa (95%CI)**	**0.558 (0.381, 0.736)**		**0.727 (0.584, 0.870)**		**0.776 (0.639, 0.914)**	
	**Serum**	**-**	43 (48)	6 (7)	37 (42)	20 (22)	52 (58)	5 (6)
		**+**	11 (12)	29 (33)	4 (4)	28 (31)	9 (10)	23 (26)
		**Kappa (95%CI)**	**0.610 (0.444, 0.775)**		**0.472 (0.303, 0.642)**		**0.649 (0.482, 0.816)**	
	**Shedding**	**-**	31 (35)	5 (6)	30 (34)	6 (7)	29 (33)	7 (8)
		**+**	23 (26)	30 (34)	11 (12)	42 (47)	32 (36)	21 (24)
		**Kappa (95%CI)**	**0.395 (0.223, 0.568)**		**0.612 (0.448, 0.777)**		**0.182 (0.012, 0.351)**	

Responders (+) and non-responders (-) for *S*. *sonnei* antigen specific IgA- and IgG-ALS were compared with responders and non-responders in the other immune categories and to vaccine shedding. Data on the main diagonal of each matrix counts the concordance number or the number of observed agreements between two measurements (+/+ and -/-) while the off-diagonal numbers counts the discordant numbers or the number of observed disagreements (+/- and -/+). For each comparison the calculated Kappa statistic is given reflecting the strength of agreement between the ALS responders and the responders in the other immune categories and shedding.

### Magnitude of individual immune responses following vaccination with 10^5^, 10^6^, and 10^7^ CFU doses of WRSs2 and WRSs3

The individual IgA and IgG responses in each category and shedding for subjects receiving 10^5^, 10^6^ and 10^7^ CFU of vaccine indicated that there were more subjects with an antigen-specific IgA-ALS/ASC/serum antibody response than with an IgG response in both groups of vaccinees. For example, among the 24 WRSs2 vaccinees, 23 subjects had positive LPS-specific IgA-ALS, 20 had IgA-ASC, 18 had positive serum IgA and 14 had fecal IgA response. In the same group of vaccinees, 17, 15 and 17 subjects each were positive for LPS-specific IgG- ALS, ASC and serum antibodies. Overall, there were more responders in the WRSs2 group than in the WRSs3 vaccinated group (Table 1). Three of the four subjects that were negative for LPS-specific IgA ASCs also lacked fecal IgA and two subjects lacked serum IgA. There were several cases in both groups of vaccinees where subjects positive for an antigen-specific IgA- or IgG-ALS response had no IgG ASC response to that antigen and vice-versa (data not shown). For example, among IgG responders in the WRSs2 group, there were 5 subjects with a positive LPS-specific IgG-ALS but <10 IgG-ASC and another 3 subjects where the reverse was true. There were also 5 subjects with ≥10 IpaB-specific IgG-ASC with no corresponding ALS response (data not shown). Compared to WRSs2, there were fewer number of responders as well as lower level of responses in the WRSs3 group of vaccinees but the associations between the different immune categories are similar (this study, 8). Although there is a high association between responders in one immune category and responders in another category as described earlier (Table 3), we noticed that the magnitude of the individual immune response in one category does not associate with a proportionate increase or decrease in the magnitude of an immune response in a second category. This was more obvious with fecal IgA responses and vaccine shedding. Six of 24 (25%) subjects in the WRSs2 vaccine group and 8 of 24 (33%) subjects in the WRSs3 vaccine group showed very low levels of shedding (CFU/gm of stool ≤45). However, the magnitude of vaccine shedding appeared to bear no proportionality to the magnitude of the other immune responses, although non-shedders and low shedders in both groups of vaccinees had lower to no immune responses in one or more categories.

## Discussion

A previous report has provided *S*. *sonnei* antigen-specific IgA/IgG serum antibodies levels, ASCs and fecal IgA responses in subjects vaccinated with WRSs2 and WRSs3 [8]. In this report, the magnitude and kinetics of the *S*. *sonnei* antigen-specific ALS responses are described showing the high level of agreement between the ALS responders and responders to the other measured immune parameters. This level of detailed analysis for a live oral *Shigella* vaccine has not been previously described. The responder frequency in this trial is based on the pre-determined definition for each immune measurement (≥4-fold rise in ELISA titers over baseline and ≥10 ASCs /10^6^ PBMCs).

The ALS assay for an oral, *virG(icsA)*-based *Shigella* vaccine candidate was initially described using culture supernatants from freshly isolated PBMCs obtained during a phase 1 trial of WRSd1, a live *S*. *dysenteriae* 1 vaccine candidate [11]. The supernatants for the ALS assay in the current study were obtained from frozen PBMCs. Along with the WRSd1 samples, placebo samples from a rifaximin study, where the antibiotic was given to subjects after challenge with a *S*. *flexneri* 2a strain, were also assayed [11,21,26]. LPS-specific IgA- and IgG-ALS and ASC responses from 50 subjects were shown to be comparable, with ALS proving to be more sensitive [11,21,26]. Although this is generally the case for IgA-ALS responses described in this study, there were some subjects in both WRSs2 and WRSs3 vaccines where antigen-specific IgA and IgG-ALS responses did not correlate with a corresponding IgA and IgG ASC response and vice-versa. Furthermore, we observed that the magnitude of an individual response in one category was not proportionate to the magnitude of another response even though there is strong agreement between responses and responders in the two categories. For example, 4 of 24 subjects in the WRSs2 group demonstrated a maximum LPS-specific IgA ALS response of 512 that corresponded to 37, 84, 148 and 108 IgA-ASCs per 10^6^ PBMCs and 32, 8, 32, and 8-fold increase in serum IgA levels from baseline, respectively. This could be partly explained by realizing what these two assays measure. The ELISPOT assay identifies the frequency of antigen-specific antibody secreting cells directly, by the binding of the secreted antibody to a membrane-bound antigen that is detected and counted as a colored spot [13,27]. In addition, each spot size represents the integration of the amount of the secreted antibody and its secretion kinetics, providing important biological information [27]. In contrast, in the ALS assay the culture supernatants obtained from the *ex vivo* antigen-free cell culture of PBMCs is enriched for the total antibodies secreted by the antibody secreting cells and has to reach an ELISA titer threshold that meets the set criteria for a response. The ELISA titer could be a reflection of a few highly active or several normal to less active plasmablasts. We realize that the number of antibody secreting cells is not as relevant as the magnitude of the antibody response and that if there is a threshold of an ALS titer associated with protection, it would be irrelevant if that titer was reached through the secretion from a few highly active plasmablasts or several less active plasmablasts.

One of the outstanding issues in *Shigella* vaccine development is the lack of a known distinct and measurable correlate of protection. Consequently, an immune correlate could vary based on the type of vaccine candidate and the route of immunization and some efforts have been directed towards defining correlates of immunity and protection [28–32]. In an earlier study with SC602, a live *S*. *flexneri* 2a vaccine candidate, 7 of 7 vaccinated subjects who were challenged with a virulent *S*. *flexneri* 2a strain, were protected against fever, moderate to severe diarrhea, dysentery and the severe symptoms of shigellosis that were seen in 7 of 8 unvaccinated control subjects [9]. LPS-specific IgA-ASCs of >45 spots per 10^6^ PBMCs along with significant serum IgA/IgG/IgM responses appeared to correlate with complete protection [9]. Those with milder symptoms after challenge had none to <45 LPS-specific IgA ASCs/10^6^ PBMCs and low serum antibodies [9]. Although this study was carried out in a limited number of subjects, the clinical data from the SC602 study indicates that protection against disease requires a threshold level of mucosal and systemic responses that could be quantified and validated by further studies in a larger group of subjects. Thus, the associations between the various immune parameters and shedding described in this report will have further relevance in an efficacy study with these oral vaccine candidates. In prior field studies with parenterally-administered O-antigen-based subunit *Shigella* vaccine candidates, high serum IgG titers were shown to correlate with vaccine efficacy in adults and children but the same vaccine candidates failed to protect infants and toddlers <3 years of age [31]. This observation has been recently confirmed using a bioglycoconjugate vaccine candidate that was administered to healthy adults in the U.S. who were subsequently challenged with a virulent *Shigella* strain [32]. The high serum IgG levels is thought to transudate over mucosal epithelial cells and inactivate the bacteria in a complement-mediated bactericidal activity [32]. Whether a similar mechanism also operates with oral *Shigella* vaccines remains to be seen.

A more refined ALS assay was recently carried out in a controlled human infection model (CHIM) with a *S*. *sonnei* virulent strain 53G using PBMCs carrying α4β7+, the gut homing integrin marker [33]. Subjects progressing to shigellosis had substantially higher LPS and IVP-specific α4β7+ ALS responses compared to subjects without shigellosis [33]. Since pre-vaccination LPS-specific IgG titers are often used in subject exclusion criteria in many *Shigella* phase 1 trials, including in the WRSs2/WRSs3 study, surprisingly the 53G CHIM study indicated that, subjects with higher LPS-specific baseline titers of serum IgA, fecal IgA and -memory B cell IgA, but not IgG, did not progress to shigellosis [33]. Due to limitation in blood volume collection, ASCs were not performed in the 53G CHIM study. In a recent workshop, a recommended list of immunoassays to be performed during a *Shigella* vaccination study included the ALS assay with α4β7 positive and negative cells to record mucosal response [34]. It remains to be determined whether an α4β7 positive ALS titer along with other immune categories of responses will also provide a correlate of protection for live oral vaccines.

Since both ALS and ASC assays originate with the same batch of mucosally-primed circulating plasmablasts, it is not surprising that a very high agreement exists between the IgA- and IgG-ALS responders/non-responders and IgA- and IgG-ASC and serum antibody responders/non-responders to LPS, IVP and IpaB in both vaccination groups. However, the relationship to shedding is worth noting since vaccine shedding is taken as a surrogate of mucosal colonization, and is directly linked to immunogenicity [9,10,35]. Low shedders or non-shedders elicit poor immune responses also documented with other live oral *Shigella* vaccine candidates [9,10,35]. However, the magnitude of shedding in each subject is not proportional to the magnitude of some of the other immune responses. This anomaly may reflect the nature of the sample collected and the timing. Detection of *Shigella* and *Shigella*-specific antibodies in stool is subjective, unpredictable and technically challenging. We noted an association between shedding and some of the antigen-specific immune responses among the WRSs3, but not WRSs2, vaccinees. Since WRSs3 lacks the *msbB2* gene that deacylates the lipid A portion of the bacterial LPS and reduces its endotoxicity, the association between the loss of the *msbB2* gene in WRSs3, its shedding in vaccines and the immune response is not clear.

The ALS assay has been used in vaccine studies of other bacterial diarrheal pathogens [36–38]. In a study of three genetically modified live ETEC strains, serum antibody responses and ALS were more predictive of a mucosal IgA response than the ASCs [36]. With an oral formalin-killed cholera vaccine, ASC responses were similar to those detected by ALS assays although current studies with cholera vaccines continue to use the ASC assay for measuring mucosal response [37,38]. In a live typhoid vaccine study, both ASC and ALS assays demonstrated 100% vaccine-specific responses similar to what is seen here with WRSs2/WRSs3 study [39–41]. A positive *S*. *typhi* LPS-specific ASC response has been associated with efficacy in field trials [41]. Although there was high concordance between the two mucosal immune assays, 15% of the subjects with *S*. *typhi* LPS-specific IgA-ASC responses were negative for IgA-ALS, a feature also seen in a number of WRSs2 and WRSs3 vaccinees. The ALS data in the *S*. *typhi* trial was in agreement with the ASC responses when the ASCs were ≥42 spots/10^6^ PBMCs [40,41].

In conclusion, the ease of performing the ALS assay in an ELISA format and the convenience of collecting, aliquoting, freezing and transporting culture supernatants, if needed, to different labs for multiple assays makes this assay more feasible over ASC assays, especially in children and in regions with limited resources. Although the ELISpot assay is a robust technique, the assay can be technically challenging with fresh or frozen PBMCs and limited by the proportion of antigen-specific ASCs in PBMCs [42]. While it is clear that there is strong agreement between antigen-specific ALS and ASC measurements, it remains to be seen in future efficacy studies with live oral *Shigella* vaccines, whether an ALS titer or a certain number of antigen-specific ASCs fulfils the role of a mucosal correlate of protection.
